# Predicting Retrograde Autobiographical Memory Changes Following Electroconvulsive Therapy: Relationships between Individual, Treatment, and Early Clinical Factors

**DOI:** 10.1093/ijnp/pyv067

**Published:** 2015-06-19

**Authors:** Donel M. Martin, Verònica Gálvez, Colleen K. Loo

**Affiliations:** Black Dog Institute, School of Psychiatry, University of New South Wales, Sydney, Australia; (Drs Martin, Gálvez, and Loo); Wesley Hospital, Sydney, Australia (Dr Loo); St George Hospital, South Eastern Sydney Health, Sydney, Australia (Dr Loo).

**Keywords:** Electroconvulsive therapy, memory, verbal fluency, orientation time

## Abstract

**Background:**

Loss of personal memories experienced prior to receiving electroconvulsive therapy is common and distressing and in some patients can persist for many months following treatment. Improved understanding of the relationships between individual patient factors, electroconvulsive therapy treatment factors, and clinical indicators measured early in the electroconvulsive therapy course may help clinicians minimize these side effects through better management of the electroconvulsive therapy treatment approach. In this study we examined the associations between the above factors for predicting retrograde autobiographical memory changes following electroconvulsive therapy.

**Methods:**

Seventy-four depressed participants with major depressive disorder were administered electroconvulsive therapy 3 times per week using either a right unilateral or bitemporal electrode placement and brief or ultrabrief pulse width. Verbal fluency and retrograde autobiographical memory (assessed using the Columbia Autobiographical Memory Interview – Short Form) were tested at baseline and after the last electroconvulsive therapy treatment. Time to reorientation was measured immediately following the third and sixth electroconvulsive therapy treatments.

**Results:**

Results confirmed the utility of measuring time to reorientation early during the electroconvulsive therapy treatment course as a predictor of greater retrograde amnesia and the importance of assessing baseline cognitive status for identifying patients at greater risk for developing later side effects. With increased number of electroconvulsive therapy treatments, older age was associated with increased time to reorientation. Consistency of verbal fluency performance was moderately correlated with change in Columbia Autobiographical Memory Interview – Short Form scores following right unilateral electroconvulsive therapy.

**Conclusions:**

Electroconvulsive therapy treatment techniques associated with lesser cognitive side effects should be particularly considered for patients with lower baseline cognitive status or older age.

## Introduction

Despite significant advances in electroconvulsive therapy (ECT) technique, memory side effects continue to be of major concern for patients and clinicians. In particular, loss of personal memories from the period prior to receiving ECT treatment (ie, retrograde autobiographical memory) is common and in some patients can persist for many months following treatment (Squire et al., 1981; Sackeim et al., 2007). Memory loss may include specific events, details about events, or thoughts and feelings experienced during the missing periods. The severity of these side effects, however, can be potentially reduced through the tailoring of ECT treatment parameters according to individual patient factors and the modification of treatment parameters during the ECT course based on early clinical indicators. Improved understanding of the relationships between individual patient, ECT treatment factors, and early clinical indicators may facilitate better management of the ECT treatment approach to minimize later cognitive side effects.

It is now well established that certain ECT treatment factors are critically related to both the severity and type of cognitive side effects experienced by patients. For example, the use of higher electrical dose (Ottosson, 1960; Sackeim et al, 1993; McCall et al, 2000; Quante et al., 2011), increased frequency of treatments (Lerer et al., 1995), and choice of stimulus parameters, including use of sine wave stimulation (Sackeim et al., 2007) and brief-pulse instead of “ultrabrief” pulse width stimulus (Sackeim et al., 2008; Loo et al., 2014; Tor et al., in press), have been demonstrated to be associated with increased cognitive side effects. In addition, the choice of ECT electrode placements is important, as montages that minimize the degree of temporal lobe stimulation, including right unilateral (RUL), have been found beneficial for reducing memory side effects (Sackeim et al., 1993; Sobin et al., 1995; Dunne and McLoughlin, 2012). This empirical research is supported by findings from a large community study that showed that these ECT treatment factors explained a large degree of variability in patient outcomes between different hospitals (Sackeim et al., 2007). Specifically, the ECT treatment factors associated with greater retrograde autobiographical memory side effects include the use of bitemporal (BT) electrode placement, longer pulse-widths, and increased number of treatments (Sackeim et al., 2007; Loo et al., 2008, 2012; Tor et al., in press).

Individual patient factors are also important for predicting cognitive outcomes. In relation to retrograde autobiographical memory side effects, identified predictors include pre-ECT global cognitive status, estimated premorbid IQ, age, and bipolar depression diagnosis (Sobin et al., 1995; Sackeim et al., 2007; Martin et al., 2013). The relative importance of these individual patient factors for predicting later side effects, however, remains unclear. Moreover, research into predictors of ECT-related cognitive side effects has found that early clinical indicators during the ECT course, for example measuring time to reorientation and early cognitive changes, are better predictors of outcomes (Sobin et al., 1995; Martin et al., 2013). Sobin et al. (1995) showed that patients who took the longest time to reorient immediately post ECT had the poorest retrograde autobiographical memory outcomes both in the week following ECT and at 2 months follow-up. This strong association was interpreted by the authors to suggest that post-ictal disorientation may indeed represent a similar phenomenon to retrograde amnesia, as both cognitive side effects have a similar temporal gradient (Daniel et al., 1987). Despite this strong evidence as an early clinical indicator for later side effects, routine measurement of time to reorientation is uncommon in clinical settings. This may in part be due to difficulties with interpretation, as longer times to reorientation following the ECT stimulus likely result from complex interactions between individual patient and ECT treatment factors. For these reasons, we aimed first to determine the relative importance of individual patient factors prior to the ECT course and second to clarify the relationships between individual patient factors, ECT treatment factors, and time to reorientation measured during the ECT course for predicting later retrograde autobiographical memory side effects.

Further, measurement of retrograde autobiographical memory side effects poses specific methodological (ie, Semkovska and McLoughlin, 2013) and pragmatic challenges (eg, need for detailed patient interviews) in clinical settings. A brief cognitive measure that can be used to monitor retrograde memory outcomes would therefore be of potential clinical benefit. Verbal fluency measures, which typically are used to assess executive functioning, are sensitive to the effects of ECT (Sackeim et al., 2008; Loo et al., 2008; Dybedal et al., 2014) and are also brief and simple to administer. Moreover, performance on verbal fluency measures similarly involves retrieval processes, which are critical to retrograde memory functioning. Memory retrieval is known to be subserved in part by the right inferior frontal gyrus (Burianova and Grady, 2007; Burianova et al., 2010), a region shown by computational modelling to be largely affected by the ECT stimulus, particularly for RUL and BT ECT (Bai et al., 2012a). As such, we hypothesized that change in verbal fluency performance across the ECT course may be associated with ECT-related changes in retrograde memory.

The first aim of this study was to determine the relative importance of individual patient factors for predicting later retrograde memory side effects. A secondary aim was then to clarify the associations between individual patient factors, ECT treatment factors and time to reorientation during the ECT course for predicting retrograde autobiographical memory outcomes. Lastly, we investigated the utility of measuring intra-individual changes in verbal fluency performance for monitoring retrograde memory side effects post ECT.

## Methods

### Participants

Participants were 74 inpatients drawn from a larger double-blind, randomized trial of ultrabrief vs brief pulse ECT (NTC00870805, RUL sample reported in Loo et al., 2014). From the larger trial, only participants for whom complete clinical and cognitive data were available were included in this analysis. Participants were treated with ECT at 2 hospitals in Australia: Wesley Hospital Kogarah and The Melbourne Clinic. Inclusion criteria were: age ≥18 years, DSM-IV Major Depressive Episode, no diagnosis of schizophrenia, schizoaffective disorder or rapid cycling bipolar disorder, no ECT in the last 3 months, no drug or alcohol abuse in the last 6 months, no past or current neurological illness or injury, score ≥24 on Mini Mental State Examination (MMSE; Folstein et al., 1975), and English proficiency sufficient to undertake cognitive testing.

All participants remained under the care of their own treating psychiatrist during the trial period, and decisions concerning the number of ECT treatments received, the decision to switch electrode placements, and concurrent medications were determined clinically by that psychiatrist.

The trial was approved by the Human Research Ethics Committees of the University of New South Wales, The Melbourne Clinic and Ramsay Healthcare. Participants were prescribed ECT by their own treating psychiatrist and gave informed consent for ECT treatment and participation in the research trial. Demographic and clinical characteristics were obtained by a psychiatrist or research psychologist.

### ECT Treatment

ECT was given 3 times per week with either a BT or RUL (d’Elia placement) electrode placement. Electrode placement was not randomized and was prescribed according to each patient’s treating psychiatrist. Participants were anaesthetized with either thiopentone (3–5mg/kg) or propofol (1–2mg/kg), followed by succinylcholine (1mg/kg). Participants were randomized to receive either brief (1.0 millisecond [ms]) or ultrabrief (0.3ms) pulse widths using a computer-generated random number sequence, stratified by electrode placement and trial site. At doses of 500 to 1000 milliCoulombs, the ultrabrief pulse width had to be increased to 0.4 to 0.7ms to achieve higher absolute electrical doses because of limitations of parameter settings. For each participant, seizure threshold (ST) was established by titration at the first ECT session. ECT treatments were given at 5×ST for brief pulse RUL ECT; 8 × ST for ultrabrief pulse RUL ECT; 1.5×ST for brief pulse BT ECT; and 3×ST for ultrabrief pulse BT ECT. Ultrabrief pulse RUL ECT was given at 8 times ST to determine whether a higher relative dosage level would result in comparable efficacy and cognitive side effects to brief-pulse RUL ECT (Loo et al., 2014). Participants were retitrated at their seventh treatment, and ECT dose was adjusted as necessary to maintain dosing at the correct suprathreshold levels.

### Cognitive Testing

A trained research psychologist blinded to treatment assignment assessed cognitive functioning prior to the ECT course and again 1 to 3 days post ECT. The cognitive tests examined in the current study were drawn from a larger battery as described previously (Loo et al., 2014). Retrograde autobiographical memory was assessed using the Columbia Autobiographical Memory Interview Short Form (AMI-SF; McElhiney et al., 2001), a standardized measure of consistency of recall previously found to be sensitive to ECT-related side effects (Sackeim et al., 2007, 2008; Loo et al., 2008). Verbal fluency (FAS) was assessed using the Controlled Oral Word Association Test (Benton and Hamsher, 1976) using standardized administration and scoring procedures. Consistency of verbal fluency performance (posttreatment/baseline) was used for the purpose of examining potential associations with the AMI-SF, which similarly has a consistency outcome measure. At baseline, estimated premorbid IQ was determined using the Wechsler Test of Adult Reading (Wechsler, 2001) and global cognitive status using the MMSE. After the third and sixth ECT treatments, time to reorientation post ECT (Sobin et al., 1995) was examined every 5 minutes for up to 60 minutes.

### Mood Assessment

Severity of participants’ depression was assessed at baseline and posttreatment (1–3 days after the last ECT) by a trained psychologist, blinded to treatment assignment, using the Montgomery-Äsberg Depression Rating Scale (Montgomery and Asberg, 1979). Remission was defined as a post ECT Montgomery-Äsberg Depression Rating Scale score <10.

### Statistical Methods

Statistical analyses were conducted using Statistical Package for the Social Sciences (SPSS) for Windows, Version 21.0 (IBM Corp., Armonk, NY) and AMOS software (v. 21, SPSS Inc., Chicago, IL). For verbal fluency consistency scores, values >1 (ie, indicating better performance at posttreatment compared with baseline) were entered as 1 in the analysis to be consistent with AMI-SF scoring. Clinical and demographic characteristics for the sample were compared across the 2 sites using independent sample *t* tests for dimensional variables and chi-squares for categorical variables. Bivariate associations between the hypothesized predictor variables were examined using Pearson’s correlations. A linear multivariate analysis was then conducted to examine predictors of AMI-SF performance at posttreatment (arcsine transformed) using the following independent variables: study site, age, gender, baseline MMSE, predicted premorbid IQ, electrode placement (RUL or BT), pulse width (ultrabrief or brief pulse width), number of ECT treatments, time to reorientation post 6 ECT (log transformed), and mood at posttreatment. Additional regression analyses were then conducted to examine predictors of time to reorientation both at post 3 ECT and at post 6 ECT using the following hypothesized variables: age, sex, diagnosis (unipolar or bipolar), previous courses of ECT (yes or no), baseline MMSE, predicted premorbid IQ, electrode placement (RUL or BT), pulse width (ultrabrief or brief pulse width), anaesthetic type (propofol or thiopentone), and treatment with benzodiazepines. Path analysis was used to model the hypothesized associations between the predictor variables identified as statistically significant in the previous regression models. Lastly, potential associations between consistency of verbal fluency, retrograde autobiographical memory changes, and other patient- and treatment-related variables were analyzed using Pearson’s correlations.

## Results

### Participants

The demographic and clinical characteristics of the participants at the 2 sites are shown in Table 1. The majority of participants in the sample (75%) received RUL ECT, and 49% of all participants received ultrabrief pulse width ECT (with either RUL or BT placement).

**Table 1.** Demographic and Clinical Variables by Study Site

**Variables**	**Site 1 (N = 14**)	**Site 2 (N = 60**)	**t/χ** ^**2**^	***P***
Age	56.4 (9.9)	47.3 (12.9)	2.49	.02
Sex (female %)	42.9%	73.3%	4.81	.03
Bipolar (%)	21.4%	26.7%	0.16	.69
Current episode duration (wk)	43.4 (51.3)	26.7 (33.4)	1.50	.14
Previous ECT (%)	57.1%	41.7%	1.10	.29
Benzodiazepines (% yes)	42.9%	46.7%	0.07	.80
Antidepressant during ECT (% yes)	85.7%	95.0%	1.55	.21
Premorbid IQ	102 (16.7)	110 (10.4)	-1.70	.11
ECT placement			1.17	.28
RUL (%)	85.7%	71.7%		
Bitemporal (%)	14.3%	28.3%		
Pulse width (% ≤ .7ms)	50.0%	48.3%	0.01	.91
Anaesthetic (%)			2.60	.11
Propofol	14.3%	36.7%		
Thiopentone	85.7%	63.3%		
Number of ECT	8.79 (2.67)	8.87 (3.18)	-0.09	.93
MMSE at baseline	27.1 (3.32)	28.6 (1.57)	-1.71	.11
Time to reorientation post ECT 3 (min)	25.4 (19.3)	20.7 (12.9)	0.88	.39
Time to reorientation post ECT 6 (min)	31.0 (18.6)	21.7 (16.0)	1.90	.06
AMI-SF consistency post ECT (%)	70.3 (16.9)	74.7 (15.5)	-0.93	.36
Verbal fluency consistency post ECT (%)	74.5 (19.8)	76.5 (23.3)	-0.30	.77
MADRS baseline	33.4 (8.42)	35.5 (5.81)	-0.92	.37
MADRS post ECT	14.6 (12.9)	20.8 (10.5)	-1.90	.06
Remission (% yes)	57.1%	13.3%	12.9	<.01

Remission was defined as MADRS score <10.

Abbreviations: AMI-SF, Columbia Autobiographical Memory Interview – Short Form; MADRS, Montgomery-Äsberg Depression Rating Scale; MMSE, Mini Mental State Examination; RUL, right unilateral.

### Predictors of Retrograde Autobiographical Memory Functioning at Posttreatment

Table 2 shows the bivariate associations between hypothesized predictor variables. Change in retrograde autobiographical memory was significantly associated with baseline global cognitive functioning, pulse width, reorientation time at post ECT 3 and 6, and consistency of verbal fluency performance. Results from the regression analysis are shown in Table 3. The regression model accounted for 32% of the variance in retrograde autobiographical memory performance at posttreatment [F(10,63 = 2.97, *P*<.01]. Time to reorientation post ECT 6 (*P* = <.01) was the only significant predictor in the model.

**Table 2.** Bivariate associations between hypothesized predictor variables of change in retrograde autobiographical memory post ECT

**Variables**	**AMI-SF Post**	**Age**	**Sex**	**RUL or BT**	**MMSE Baseline**	**Pred IQ**	**Pulse Width**	**Number of ECT**	**Reorient** **ECT 3**	**Reorient ECT 6**	**MADRS Post**	**Consist VF**
AMI-SF post	1.0											
Age	-.03	1.0										
Sex	-.04	-.15	1.0									
RUL or BT	-.08	-.05	-.12	1.0								
MMSE baseline	.31**	-.09	.07	-.03	1.0							
Pred IQ	.18	-.02	-.04	.08	.38**	1.0						
Pulse width	-.32**	.05	.02	.02	-.20	-.20	1.0					
Number of ECT	-.17	-.12	-.02	-.23	-.13	-.01	-.11	1.0				
Reorient ECT 3	-.43**	-.02	.01	.23*	-.41**	.04	.37**	.10	1.0			
Reorient ECT 6	-.45**	.37**	-.07	.15	-.42**	-.04	.35**	.14	.70**	1.0		
MADRS Post	-.14	-.09	.14	.09	-.05	.00	-.14	.02	.01	.01	1.0	
Consist VF	.44**	.03	-.14	.16	.02	-.09	-.09	-.09	-.26*	-.18	.04	1.0

Values shown are Pearson’s r. **P < .01, *P < .05.

Abbreviations: AMI-SF, Autobiographical Memory Interview – Short Form; BT, bitemporal; consist VF, consistency of verbal fluency score; ECT, electroconvulsive therapy; MADRS Post, Montgomery Asberg Depression Rating Scale, measured at end of the ECT course; MMSE, Mini Mental State Examination, Pred IQ, predicted premorbid IQ; Reorient 3 and Reorient 6, time to reorientation immediately after the third and sixth ECT treatment; RUL, right unilateral.

**Table 3.** **Predictors of Retrograde Autobiographical Memory Consistency Score at Post** treatment

**Variables**	**β**	***P***
Site	.07	.56
Age	.13	.29
Sex	-.08	.48
Baseline MMSE	.07	.56
Predicted premorbid IQ	.04	.72
Electrode placement (RUL or BT)	-.06	.58
Pulse width (UB = 1, BP = 2)	-.18	.14
Number of ECT treatments	-.12	.33
Time to re-orientation post ECT 6	-.38	<.01**
MADRS post treatment	-.15	.19

**P < .01.

Abbreviations: BP, brief pulse; BT, bitemporal; MADRS, Montgomery-Äsberg Depression Rating Scale; MMSE, Mini Mental State Examination; RUL, right unilateral; UB, ultrabrief.

### Predictors of Time to Reorientation at Post ECT 3 and Post ECT 6

Results from the regression models are shown in Table 4. At post ECT 3, the regression model accounted for 45% of the variance in time to reorientation [F(10,63) = 5.05, *P*<.001]. Baseline cognitive status, predicted premorbid IQ, electrode placement, pulse width, and anaesthetic type were all significant predictors in the model (*P*<.05). At post ECT 6, the regression model accounted for 42% of the variance [F(10,63) = 4.63, *P*<.001]. In this model, only age, baseline cognitive status, and pulse width were significant predictors.

**Table 4.** Predictors of Time to Reorientation at Post ECT 3 and Post ECT 6

**Variables**	**Post ECT 3**		**Post ECT 6**	
	**β**	***P***	**β**	***P***
Age	-.06	.57	.30	<.01**
Sex	.02	.82	-.09	.39
Previous ECT (yes or no)	.04	.41	.02	.82
Diagnosis (unipolar or bipolar)	.07	.49	.10	.32
Baseline MMSE	-.28	.01*	-.24	.04*
Predicted premorbid IQ	.22	.04*	.05	.63
Electrode placement (RUL = 1, BT = 2)	.22	.03*	.08	.43
Pulse width (UB = 1, BP = 2)	.33	<.01**	.34	<.01**
Anaesthetic (Thio = 1, Prop = 2)	-.27	<.01**	-.17	.10
Benzodiazepine (yes or no)	.15	.16	.09	.40

**P < .01, *P < .05.

Abbreviations: BP, brief pulse,; BT, bitemporal; MMSE, Mini Mental State Examination, Prop, propofol; RUL, right unilateral; Thio, thiopentone; UB, ultrabrief.

### Hypothesized Path Model

**Figure 1.**
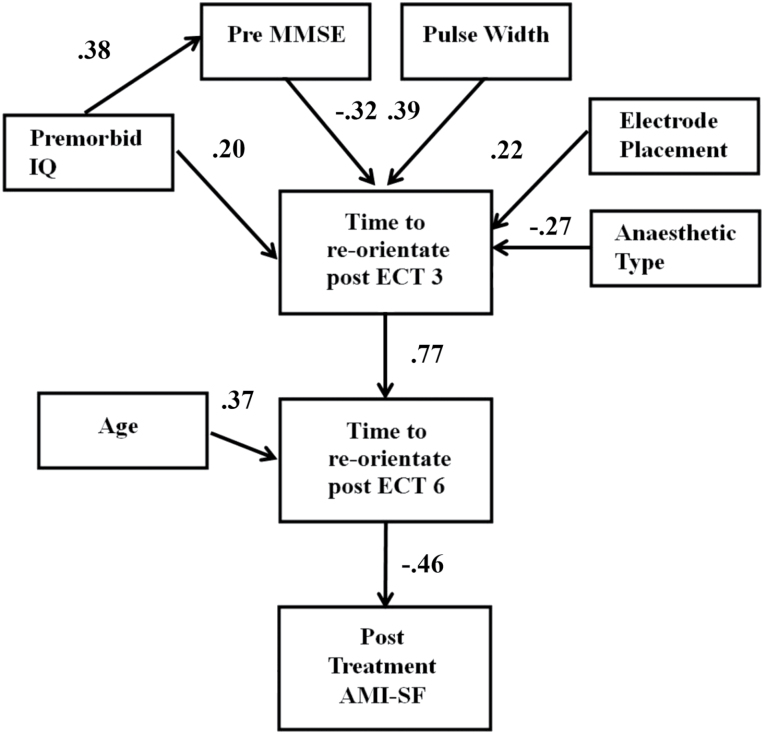
Hypothesized path model. Solid lines indicate statistically significant paths. AMI-SF, Columbia Autobiographical Memory Interview-Short Form; MMSE, Mini Mental State Examination.

Based on the results of the linear regression models, a hypothesized model of the associations between the predictor factors across the ECT course was examined using path analysis (Figure 1). This model fit the data well [χ^2^ = 25.1 (26), *P*=.51, CFI = 1.00, RMSEA = 0.00]. As can be seen in the model, predicted premorbid IQ was found to be both associated with increased global baseline functioning (a predictor of shorter time to reorientation post ECT 3) and, unexpectedly, positively associated with longer time to reorientation at post ECT 3. Older age was additionally a significant individual predictor in the model and was found to be associated with longer time to reorientation later in the ECT course (ie, at post ECT 6 but not post ECT 3). At posttreatment, greater changes in retrograde autobiographical memory were best predicted by time to reorientation post ECT 6.

### Consistency of Verbal Fluency for Monitoring Retrograde Memory Side Effects

**Figure 2.**
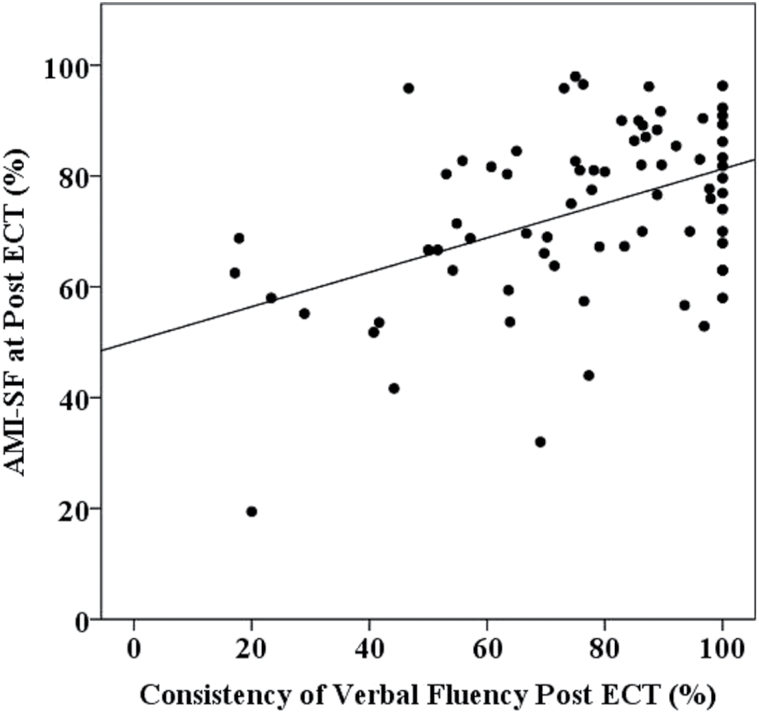
Association between consistency of verbal fluency and retrograde autobiographical memory performance following right unilateral (RUL) electroconvulsive therapy (ECT).

The associations between consistency of verbal fluency performance, retrograde autobiographical memory changes, and patient and treatment variables are shown in Table 2. There was a significant association between consistency of verbal fluency and retrograde autobiographical memory performance. Consistency of verbal fluency performance was not associated with the same patient and treatment predictors as retrograde autobiographical memory, indicating that it is not a proxy measure. Based on the magnitude of the association with electrode placement, bivariate associations between this outcome measure and the different electrode placements were analyzed separately. These analyses showed a significant moderate association between consistency of verbal fluency performance and retrograde autobiographical memory performance with RUL (r = .55, *P* <.001, N = 55) but not with BT ECT (r = .11, *P* = .67, N = 19). Figure 2 shows the association between consistency of verbal fluency and retrograde autobiographical memory performance following RUL ECT.

## Discussion

Retrograde autobiographical memory loss is often the side effect of most concern to patients receiving ECT treatment. Strategies that facilitate identification of patients most likely to develop these side effects before significant deficits have occurred and enable adjustment of ECT treatment during the course (eg, wider spacing of treatments) may therefore help to reduce overall rates of retrograde amnesia. This study found that monitoring of reorientation time during the ECT course was a greater predictor of retrograde autobiographical memory changes at post ECT than other patient and treatment factors that are mostly established at the outset of treatment. Results further showed that measuring changes in consistency of verbal fluency performance across the ECT course may be useful for monitoring retrograde memory changes following RUL ECT.

Time to reorientation in this study was measured at 2 time points, at post ECT 3 and again at post ECT 6, and individual and ECT treatment factors were investigated as predictors at both time points. The finding that time to reorientation was the strongest predictor for changes in retrograde autobiographical memory post ECT is consistent with previous research (Sobin et al., 1995). This suggests that measurement of reorientation time during the ECT course may be a useful strategy for identifying patients who are on the trajectory to significant retrograde autobiographical memory changes at the end of the ECT course. Further research should therefore examine the clinical utility of monitoring of reorientation times early in the ECT course (ie, from the first treatment), focusing both on absolute times and the significance of changes over the treatment course, with the aim of developing guidelines to help clinicians identify patients at particular risk for developing retrograde autobiographical memory side effects.

Previous research has identified both baseline cognitive status and predicted premorbid IQ as important predictors of cognitive side effects following ECT (Sobin et al., 1995; Sackeim et al., 2007; Martin et al., 2013). The current results both confirm and extend these findings by showing that they are also important predictors of time to reorientation. Baseline cognitive status as measured by the MMSE, a brief cognitive screening tool commonly used in older populations (Shulman et al., 2006), was found to be the most relevant, with patients with higher scores showing shorter times to reorientation. Higher predicted premorbid IQ was further found associated with higher MMSE scores, consistent with previous research showing IQ and education effects on MMSE scores (Spering et al., 2012; Gontkovsky, 2014). Baseline cognitive status, as measured using the MMSE, could therefore be interpreted as a proxy for cognitive reserve. Cognitive reserve is a concept that refers to the extent to which an individual’s experiential (eg, educational attainment) and neurobiological factors (eg, genetics, brain anatomy) interact to protect against pathological changes within the brain (Stern, 2002). Patients with higher cognitive reserve have previously been shown to have less anterograde memory side effects early in the ECT course (Legendre et al., 2003). Higher baseline cognitive status (measured using the modified MMSE) has also been shown to be protective against retrograde autobiographical memory side effects post ECT (Sobin et al., 1995). Taken together, these results suggest that higher cognitive reserve may reduce later retrograde autobiographical memory changes through protecting against disorientation early in the ECT treatment course.

In contrast, results showed that higher predicted premorbid IQ as estimated using a reading test was predictive of longer times to reorientation. Predicted premorbid IQ is commonly used as an indicator of cognitive reserve (Richards and Deary, 2005) and has previously been found to be associated with less retrograde autobiographical memory side effects in larger samples (Sackeim et al., 2007; Martin et al., 2013). This unexpected result therefore cautions against the use of simple reading tests alone as a proxy for cognitive reserve in clinical settings. Future investigation into possible combined indicators of cognitive reserve for ECT clinical use that, for example, take into account baseline cognitive status and other common proxy measures (eg, education level or occupational attainment) may allow for greater reliability for detecting patients at risk for greater side effects.

Older age was identified as a risk factor for longer reorientation times with increased ECT treatments. This finding is consistent with previous research showing relatively high rates of disorientation and confusion following ECT in elderly patients (Benbow, 1989). Research into the cognitive side effects in older adults following ECT, however, has been mixed. Sackeim et al. (2007), in a large community sample, for example, found older age to be a predictor for greater side effects in global cognition, reaction time, sustained attention, and anterograde learning, but not retrograde autobiographical memory, both following ECT and at 6 month follow-up. In contrast, other studies have not found older age to be associated with greater cognitive side effects, or instead greater cognitive improvement, using a variety of neuropsychological measures both during and following a course of ECT (Martin et al., 2013; Dybedal et al., 2014; Verwijk et al., 2014). It is possible that differences in ECT treatment technique may account for this discrepancy in findings between studies. The Sackeim et al. (2007) study was conducted in 7 different sites that varied widely in ECT treatment technique, resulting in varying cognitive outcomes between hospitals. In other studies (ie, Martin et al., 2013; Dybedal et al., 2014; Verwijk et al., 2014), however, patients received ECT treatment associated with lesser cognitive side effects (eg, RUL ECT, ultrabrief pulse width, treatment twice a week). Thus, older age may be associated with increased vulnerability for cognitive side effects, though only with particular ECT techniques. The current finding that older age was associated only with increased time to reorientation later in the ECT course (at post ECT 6 but not at post ECT 3) therefore lends support for the view that ECT treatment techniques associated with lesser cognitive impairment, as well as greater spacing of treatments, should be considered for older patients to minimize later retrograde autobiographical memory side effects.

Treatment factors found associated with longer times to reorientation early in the treatment course (ie, at post ECT 3) included the use of bitemporal electrode placement and longer pulse-width. These treatment factors have previously been associated with greater cognitive side effects, particularly for retrograde autobiographical memory (Sackeim et al., 1993, 2007; Sobin et al., 1995; Loo et al., 2008, 2014; Tor et al., in press). The current results therefore extend this previous work by additionally showing their relevance to the duration of time to reorientation early in the ECT course.

Type of anaesthetic used was additionally identified as an important predictor of time to reorientation, with shorter times found with the use of propofol compared with thiopentone. Previous research has shown faster times to emergence from anaesthesia with propofol compared with thiopentone, as measured by faster time to respond to verbal commands immediately post ECT (Lihua et al., 2014) as well as better cognitive performance in the immediate period following recovery from ECT (Butterfield et al., 2004). However, these findings may simply reflect the shorter half-life of propofol (Coolong et al., 2003). These results highlight the importance of taking the anaesthetic agent into account when interpreting reorientation times.

Measurement of retrograde autobiographical memory changes due to ECT is often impractical in clinical settings, leading to the need for alternative methods for monitoring these side effects. We hypothesized that change in verbal fluency performance would predict changes in retrograde memory performance across the ECT course because of both outcomes similarly relying on retrieval processes. Indeed, greater changes in consistency of verbal fluency performance were found to be significantly associated with greater changes in retrograde autobiographical memory but only with RUL ECT. Interestingly, across the entire sample, change in consistency of verbal fluency performance was not associated with the same predictors (ie, pulse width and baseline cognitive status) as change in retrograde autobiographical memory, unlike time to reorientation. This therefore suggests different shared neural substrates underlying both associations. While the association between time to reorientation and retrograde memory changes may reflect shared effects of ECT on primarily integrity of medial temporal structures and hippocampal functioning as previously proposed (Sobin et al., 1995; Sackeim et al., 2000), the association between consistency of verbal fluency and retrograde memory changes with RUL ECT, however, may instead reflect shared right inferior frontal lobe dysregulation. In support of this interpretation are the results of computer modelling of the ECT stimulus showing relatively greater bilateral medial temporal lobe stimulation with BT ECT (Bai et al., 2012a) and maximal brain activation of the right inferior frontal lobe independent of pulse width with RUL ECT (Bai et al., 2012b).

Bifrontal ECT, which has previously been shown to be associated with reduced cognitive side effects in some (Bailine et al., 2000) but not all studies (Dunne and McLoughlin, 2012), was not examined in this study. We note, however, that use of the BT and RUL electrode placements are the more commonly used forms of ECT worldwide; thus, the current findings are readily generalizable to most clinical settings. Note that in this study ultrabrief RUL ECT was given at a relatively higher level relative to threshold compared with some other studies of ultrabrief RUL ECT (eg, Sackeim et al., 2008), which may potentially have affected cognitive outcomes. Notwithstanding, even with use of this higher dosage method there was still an advantage found favoring ultrabrief pulse width over brief pulse width for reduced time to reorientation following ECT, consistent with previous studies (Tor et al., in press). A further consideration is that there was a difference between sites in relation to mood outcomes, with one of the sites found to have a low remission rate. Mood outcomes, however, were not found to be associated with the cognitive outcomes analyzed in the study, ie, the main findings of this study. The difference in remission rates between sites may be related to demographic differences between the patients treated at the respective hospitals (eg age).

In summary, the current results provide further support for monitoring of time to reorientation early during the ECT course for the detection of patients who are more vulnerable to retrograde autobiographical memory side effects following ECT. Further, our results highlighted the utility of assessing baseline cognitive status (ie, cognitive reserve) for the detection of patients who are more vulnerable to ECT-related side effects. The finding that older age was associated with longer times to reorient later during the ECT course (ie, post ECT 6) provided support for the view that for these patients, ECT treatment factors associated with lesser cognitive side effects (ie, ultrabrief ECT, RUL electrode placement, wider spacing of treatments) should be considered to minimize later retrograde memory side effects. Lastly, we report the novel finding that a simple and quick-to-administer cognitive measure (consistency of verbal fluency performance) may be useful for monitoring retrograde amnesia side effects following RUL ECT. Future research is therefore warranted to replicate the current findings and confirm the utility of this measure.

## Statement of Interest

Colleen Loo has received honoraria from Astra Zeneca and Mecta for presenting at an academic conference and international course sponsored by these companies.
